# Association of deep learning–derived epicardial fat volume with target organ damage in subjects with nonobstructive coronary artery disease

**DOI:** 10.1186/s44348-025-00062-5

**Published:** 2025-12-25

**Authors:** Moon Young Kim, Hack-Lyoung Kim, Eun Ju Chun, Ye Ra Choi, Kwang Nam Jin

**Affiliations:** 1 Department of Radiology, Seoul Metropolitan Government Seoul National University Boramae Medical Center, Seoul National University College of Medicine, Seoul, Korea; 2 Department of Internal Medicine, Seoul Metropolitan Government Seoul National University Boramae Medical Center, Seoul National University College of Medicine, Seoul, Korea; 3 Department of Radiology, Seoul National University Bundang Hospital, Seoul National University College of Medicine, Seongnam, Korea

**Keywords:** Epicardial adipose tissue, Deep learning, X-ray computed tomography, Left ventricular dysfunction, Echocardiography

## Abstract

**Background:**

Epicardial fat exerts both protective and deleterious effects on organs through diverse cytokine-mediated pathways. This study aimed to investigate computed tomography (CT)-based indexed epicardial fat volume (EFVi) in association with target organ damage parameters.

**Methods:**

The prospectively enrolled cohort of 75 patients with nonobstructive coronary artery disease underwent electrocardiogram-gated CT and was evaluated for target organ damage parameters: estimated glomerular filtration rate, proteinuria, echocardiographic septal e′ velocity, E/e′ and tricuspid regurgitation velocity, brachial-ankle pulse wave velocity, and ankle-brachial index. EFVi was measured from semiautomated 3D segmentation of electrocardiogram-gated CT. Partial correlation, multiple linear regression, and receiver operating characteristic (ROC) analyses were conducted.

**Results:**

Age and EFVi showed moderate positive linear correlation (r = 0.567, P < 0.001). After adjusting for age, EFVi was significantly correlated with the septal e′ velocity (r = − 0.489, P < 0.001) and E/e′ (r = 0.256, P = 0.034), but not with other target organ damage parameters (P > 0.05). Multiple linear regression analysis showed that the correlations of the EFVi with the septal e′ velocity (β = –0.0003, P = 0.007) and E/e′ (β = 0.0606, P = 0.024) remained significant after adjusting for potential confounders. ROC analysis identified optimal EFVi thresholds: 95.78 cm^3^/m^2^ for reduced septal e' velocity (area under the ROC curve [AUC], 0.750; sensitivity, 88.2%; specificity, 56.8%) and 91.68 cm^3^/m^2^ for elevated E/e' (AUC, 0.692; sensitivity, 71.4%; specificity, 64.8%).

**Conclusions:**

EFVi was related to left ventricular diastolic function more than other target organ damage parameters, including renal function and arterial stiffness, which suggests that the epicardial fat may have a role in the pathogenesis of left ventricular diastolic dysfunction.

**Supplementary Information:**

The online version contains supplementary material available at 10.1186/s44348-025-00062-5.

## Background

Epicardial adipose tissue (EAT), covering approximately 80% of the heart's surface, is recognized not only as a passive fat depot but as an active organ involved in the metabolic and inflammatory processes of the cardiovascular system [1]. This visceral fat depot represents a significant source of biomolecules, cytokines, and hormones, exerting both paracrine and vasocrine effects on cardiovascular tissues [2]. Understanding EAT dynamics has become increasingly important given its dual role in cardiovascular pathophysiology, potentially offering both protective and detrimental effects depending on physiological conditions [3]. Among various imaging modalities, including echocardiography, thoracic computed tomography (CT), and cardiovascular magnetic resonance imaging, electrocardiogram (ECG)-gated coronary CT angiography (CCTA) is considered the noninvasive gold standard for EAT volume measurement due to its exceptional spatial resolution and reproducibility [4].

The association between increased EAT volume and heightened risks of metabolic syndrome and cardiovascular diseases has emerged as a focal point of contemporary research, highlighting EAT's pivotal role in metabolic dysregulation and cardiovascular pathologies [5]. EAT thickness demonstrates strong correlations with key markers of metabolic syndrome, particularly waist circumference and prediabetic states, suggesting its potential utility as a biomarker for metabolic and cardiovascular risk stratification [6]. Furthermore, EAT volume has been linked to the distribution and severity of coronary artery disease (CAD), indicating its value in identifying individuals at risk of premature CAD and significant coronary stenosis [7].

Recent investigations have extended beyond traditional cardiovascular risk factors, proposing EAT as both a surrogate marker and therapeutic target for target organ damage [8]. EAT's associations with established target organ damage parameters, including ankle-brachial index (ABI), brachial-ankle pulse wave velocity (baPWV), and carotid intima-media thickness, underscore its potential in predicting vascular and arterial health deterioration [9, 10]. Additionally, EAT has been suggested as a valuable parameter for detecting early diastolic dysfunction, further illustrating its clinical significance in cardiovascular risk assessment [11, 12].

Despite compelling evidence linking EAT with various cardiovascular risk factors and target organ damage parameters, inconsistencies in the literature regarding relationships between target organ damage parameters and EAT thickness measured by transthoracic echocardiography (TTE) persist [5, 7, 13]. Moreover, comprehensive analyses examining EAT's association with a broad spectrum of target organ damage parameters remain limited, highlighting a significant gap in current research. This gap is particularly pronounced in studies focusing on Asian populations, where data on EAT's relationship with target organ damage parameters are scarce.

Considering this context, our study aimed to investigate the association between deep learning–derived epicardial fat volume on ECG-gated CCTA and multiple target organ damage parameters in subjects with nonobstructive CAD. By evaluating indexed epicardial fat volume (EFVi) as a body size–adjusted metric, we sought to determine how epicardial fat relates to renal function, arterial stiffness, and, in particular, left ventricular (LV) diastolic dysfunction within an Asian cohort [14, 15].

## Methods

### Study design and population

This single-center, prospective observational study was conducted at Seoul Metropolitan Government Seoul National University Boramae Medical Center between February 2017 and March 2021. The study protocol was approved by the Institutional Review Board and Ethics Committee of Seoul Metropolitan Government Seoul National University Boramae Medical Center (No. 30–2021-95), and the study was conducted in accordance with the principles of the Declaration of Helsinki. Written informed consent was obtained from all participants prior to study enrollment.

**Fig. 1:**
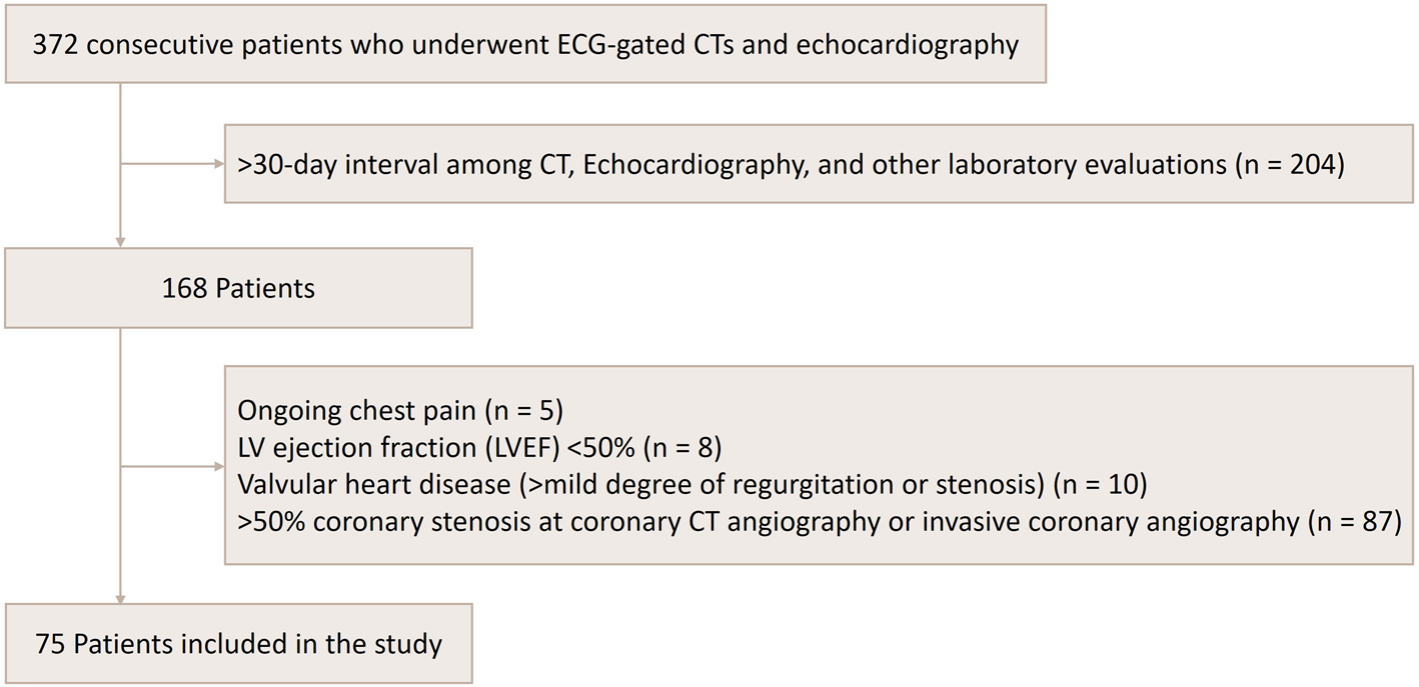
Flowchart of study population enrollment. The exclusion criteria were not mutually exclusive, and some patients met more than one criterion. ECG, electrocardiogram; CT, computed tomography; LVEF, left ventricular ejection fraction

Figure 1 illustrates the study population enrollment. The study population comprised consecutive patients who underwent ECG-gated CCTA and TTE for EFV measurement and cardiac evaluation. Initially, 372 consecutive patients were screened for enrollment. Patients were excluded if they met any of the following criteria: (1) > 30-day interval among CT, echocardiography, and other laboratory evaluations (n = 204); (2) ongoing chest pain at the time of examination (n = 5); (3) LV ejection fraction (LVEF) < 50% (n = 8); (4) valvular heart disease with more than mild degree of regurgitation or stenosis (n = 10); and (5) significant CAD with ≥ 50% coronary stenosis on CCTA or invasive coronary angiography (n = 87). After applying these exclusion criteria, 75 patients with insignificant CAD were included in the final analysis. Clinical variables such as age, sex, body mass index (BMI), mean brachial systolic and diastolic blood pressures, diabetes mellitus, dyslipidemia, smoking status, and concomitant medications (e.g., statins, calcium channel blockers, renin-angiotensin system blockers, and beta blockers) were investigated. Additionally, laboratory findings including mean estimated glomerular filtration rate (eGFR), hemoglobin A1c (HbA1c), and total cholesterol were recorded.

### Target organ damage parameters

Multiple target organ damage parameters were systematically evaluated to assess cardiovascular risk and organ dysfunction. Renal function was assessed using two key parameters: eGFR calculated by the modification of diet in renal disease equation, and proteinuria measured as albumin (μg) to creatinine (mg) ratio (ACR) from random urine samples.

Arterial stiffness and peripheral vascular function were evaluated using baPWV and ABI [16, 17]. These measurements were obtained using a volume-plethysmographic apparatus (VP-1000, Colin Co Ltd). The phonogram and pulse volume waveform were recorded with cuffs placed around both the brachia and the ankles. The ABI was calculated by dividing the ankle pressure by the brachial artery pressure.

Cardiac diastolic function was assessed using 2D TTE performed with commercially available devices (Sequoia, Siemens Medical Solutions; or Vivid 7, GE Medical Systems) according to current guideline recommendations [18]. Septal e′ velocity and E/e′ ratio were measured as key indicators of LV diastolic function [18]. Pulsed wave Doppler examination was performed at the tip of the mitral leaflets to measure the peak early mitral inflow velocity during early diastole (E), late diastole (A), and the deceleration time. Color-coded tissue Doppler imaging was performed on the apical four-chamber view to calculate the early velocity (e′) at the septal mitral annulus. Tricuspid regurgitation (TR) velocity was obtained using continuous-wave Doppler from the apical four-chamber view at the tricuspid valve.

### EFV measurement

All patients underwent retrospectively ECG-gated CT scans using a 128-slice CT scanner (Ingenuity, Philips Medical Systems). We measured the temporal interval between CT, echocardiography, and laboratory examinations to ensure consistency of measurements.

**Fig. 2:**
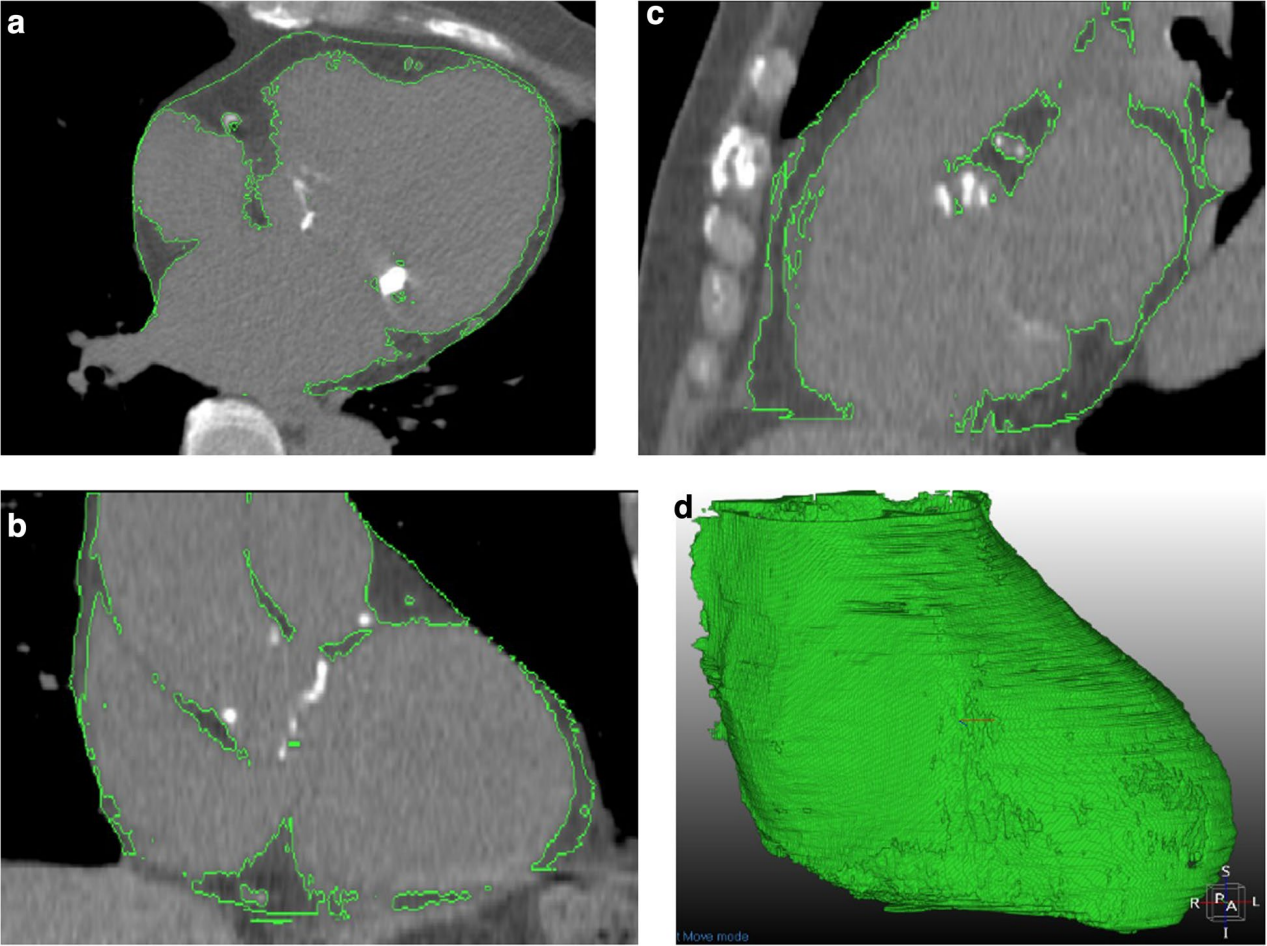
Semiautomated segmentation of epicardial fat volume (EFV) on cardiac Computed Tomography. Epicardial fat was segmented on (**A**) axial, (**B**) coronal, and (**C**) sagittal planes using deep learning–based software (green contours) and (**D**) reconstructed into a 3D volume-rendering image. EFV was measured by including voxels with attenuation between –190 and –30 Hounsfield units, which were defined as adipose tissue

Epicardial fat was defined as the adipose tissue located between the myocardium and the visceral pericardium. Initial automated segmentation was performed using 3D U-NET software MEDIP (MEDICAL IP Co Ltd) with deep learning approaches for accurate fat segmentation (Fig. 2). Following this automated segmentation, each region of interest was minimally refined at the boundaries, and possible internal holes were filled by two experienced cardiothoracic radiologists (MYK and KNJ) to ensure accuracy and consistency.

The anatomical boundaries for epicardial fat measurement were carefully defined. The upper limit of the pericardium was set at the bifurcation of the pulmonary trunk, while the lower boundary extended to the inferior aspect of the pericardial sac. All voxels with attenuation values between –190 and –30 Hounsfield units (HU) were identified as adipose tissue within the traced epicardial space.

To account for individual body size variations, EFVi was calculated as the measured EFV divided by body surface area (BSA). EFV and EFVi were expressed in cubic centimetres (cm^3^) and cubic centimeter per square meter (cm^3^/m^2^), respectively. The BSA was calculated using the Mosteller equation:

$$\text{BSA} ({m}^{2})=\sqrt{\frac{(height in cm)\times (weight in kg)}{\text{3,600}}}$$

### Statistical analysis

Statistical analyses were performed using IBM SPSS ver. 26 (IBM Corp) and Rex ver. 3.0.3 (RexSoft Inc). Continuous variables are presented as mean ± standard deviation, while categorical variables are presented as numbers and percentages. The normal distribution of continuous variables was assessed using the Shapiro–Wilk test.

Pearson correlation analysis was conducted to examine the association between EFVi and various target organ damage parameters [15]. To control for the potential confounding effect of age, partial correlation analysis was performed with age as the control variable [19]. The strength of correlations was interpreted according to conventional criteria: weak (r < 0.3), moderate (r = 0.3 to 0.7), and strong (r > 0.7).

Multivariable linear regression analysis was performed to investigate which target organ damage parameters were independently associated with EFVi. Potential confounding variables including age, BMI, hypertension, diabetes mellitus, smoking status, and eGFR were included in the regression models as adjustment factors, considering the established associations between these cardiovascular risk factors and target organ damage [16]. Model fit was assessed using adjusted R^2^. Regression coefficients (β) with 95% confidence intervals (CIs) and P-values were reported.

Receiver operating characteristic (ROC) curve analyses were conducted to evaluate the diagnostic performance of EFVi in predicting specific target organ damage thresholds. The optimal cutoff values were determined using the Youden index, which maximizes the sum of sensitivity and specificity. Areas under the ROC curves (AUCs) were calculated to assess discriminative ability, with values > 0.7 considered acceptable diagnostic performance. The 95% CIs were estimated using stratified bootstrap resampling (1,000 replicates).

A two-tailed P-value of less than 0.05 was considered statistically significant for all analyses. All statistical tests were performed with appropriate assumptions verified, and missing data were handled using listwise deletion.

## Results

### Baseline characteristics of the study population

The baseline clinical characteristics of the 75 enrolled participants are summarized in Table 1. The mean age was 57.1 ± 13.6 years, and 39 (52.0%) were male. The average BMI was 26.1 ± 4.2 kg/m^2^. Mean brachial systolic and diastolic blood pressures were 123.5 ± 15.7 and 73.2 ± 10.2 mmHg, respectively. The most common cardiovascular risk factors were dyslipidemia (22.7%), hypertension (21.3%), and diabetes mellitus (13.3%). Concomitant medications included statins (30.7%), calcium channel blockers (16.0%), renin-angiotensin system blockers (16.0%), and β-blockers (13.3%).

**Table 1:** Baseline clinical characteristics of study population

Characteristic	Value (*n* = 75)
Age (yr)	57.1 ± 13.6
Sex	
Male	39 (52.0)
Female	36 (48.0)
Body mass index (kg/m^2^)	26.1 ± 4.2
Brachial systolic blood pressure (mmHg)	123.5 ± 15.7
Brachial diastolic blood pressure (mmHg)	73.2 ± 10.2
Cardiovascular risk factor	
Hypertension	16 (21.3)
Diabetes mellitus	10 (13.3)
Dyslipidemia	17 (22.7)
Current smoker	6 (8.0)
Concomitant medication	
Antiplatelet agent	9 (12.0)
Renin-angiotensin system blocker	12 (16.0)
β-Blocker	10 (13.3)
Calcium channel blocker	12 (16.0)
Statin	23 (30.7)
Maximal interval among examinations^a^ (day)	5.6 ± 8.4
Laboratory finding	
White blood cell count (/μL)	6,437 ± 2,215
Hemoglobin (g/dL)	13.9 ± 1.6
HbA1c (%)	6.2 ± 1.2
Fasting blood glucose (mg/dL)	120.5 ± 38.5
eGFR (mL/min/1.73 m^2^)	100.6 ± 33.4
Total cholesterol (mg/dL)	181.0 ± 33.7
LDL cholesterol (mg/dL)	104.9 ± 39.4
HDL cholesterol (mg/dL)	51.3 ± 12.9
Triglyceride (mg/dL)	136.1 ± 73.8
C-reactive protein (mg/dL)	0.28 ± 0.43
Urine ACR (μg/mg)	55.7 ± 177.0
Vascular function test	
baPWV (cm/sec)	1,430 ± 264
ABI	1.15 ± 0.08

Values are presented as number (%) or mean ± standard deviation

ABI, ankle-brachial index; ACR, albumin to creatinine ratio; baPWV, brachial-ankle pulse wave velocity; eGFR, estimated glomerular filtration rate; HbA1c, hemoglobin A1c; HDL, high-density lipoprotein; LDL, low-density lipoprotein

^a^Each patient’s maximal interval among computed tomography, echocardiography, and laboratory and vascular function tests

The mean interval among CT, echocardiography, and laboratory evaluations was 5.6 ± 8.4 days. Laboratory findings showed a mean eGFR of 100.6 ± 33.4 mL/min/1.73 m^2^, HbA1c of 6.2% ± 1.2%, and total cholesterol of 181.0 ± 33.7 mg/dL. The mean baPWV was 1,430 ± 264 cm/sec, and the ABI was 1.15 ± 0.08.

### CT and echocardiographic parameters

Table 2 presents the CCTA-derived epicardial fat measurements and echocardiographic findings. The mean EFV was 155.6 ± 44.7 cm^3^, and the EFVi, adjusted for BSA, was 91.4 ± 28.6 cm^3^/m^2^. The mean LVEF was preserved at 60.1% ± 4.6%. The average LV end-diastolic dimension was 50.0 ± 6.6 mm, and the deceleration time of early mitral inflow velocity was 200.0 ± 54.6 ms. The mean E/A ratio was 0.95 ± 0.37, while the mean septal e′ velocity was 6.5 ± 2.1 cm/sec and the E/e′ ratio was 10.5 ± 3.3.

**Table 2:** CT and echocardiographic parameters of study population

Parameter	Value (n = 75)
CT parameter	
EFV (cm^3^)	155.6 ± 44.7
EFVi (cm^3^/m^2^)	91.4 ± 28.6
Echocardiographic parameter	
LVEF (%)	60.1 ± 4.6
LV end-diastolic dimension (mm)	50.0 ± 6.6
Deceleration time (msec)	200.0 ± 54.6
E/A	0.95 ± 0.37
Septal e′ velocity (cm/sec)	6.5 ± 2.1
E/e′	10.5 ± 3.3
TR velocity (m/sec)	2.27 ± 0.41

Values are presented as mean ± standard deviation

CT, computed tomography; EFV, epicardial fat volume; EFVi, indexed epicardial fat volume; LV, left ventricular; LVEF, left ventricular ejection fraction; TR, tricuspid regurgitation

### Association between EFVi and various target organ damage parameters

Partial correlation analysis was performed to examine relationships between EFVi and target organ damage parameters while controlling for age as a confounding variable. As illustrated in Supplementary Fig. 1, there was a significant positive linear correlation between age and EFVi. Pearson correlation analysis revealed a moderate correlation coefficient (r = 0.567, P < 0.001), indicating that EFVi tended to increase with advancing age.

As presented in Table 3, EFVi demonstrated significant correlations with echocardiographic parameters of LV diastolic function. EFVi showed a significant negative correlation with septal e′ velocity (r = − 0.489, P < 0.001), indicating that higher EFV was associated with reduced early diastolic tissue velocity. Conversely, EFVi exhibited a significant positive correlation with the E/e′ ratio (r = 0.256, P = 0.034), suggesting an association between increased EFV and elevated LV filling pressures.

**Table 3:** Partial correlation analysis and multivariable analysis between EFVi and target organ damage parameters

Parameter	Partial correlation analysis		Multivariable analysis	
	r	*P*-value	β (95% CI)	*P*-value
Septal e′ velocity	–0.489	< 0.001^*^	–0.0003 (–0.0005 to –0.0001)	0.007^*^
E/e′	0.256	0.034^*^	0.0606 (0.0093 to 0.1120)	0.024^*^
TR velocity	–0.026	0.877		
eGFR	0.120	0.308		
Urine ACR	–0.131	0.413		
baPWV	0.145	0.218		
ABI	0.167	0.153		

EFVi was significantly associated with septal e′ velocity and E/e′ ratio in partial correlation and multivariable linear regression analysis

ABI, ankle-brachial index; ACR, albumin to creatinine ratio; baPWV, brachial-ankle pulse wave velocity; CI, confidence interval; eGFR, estimated glomerular filtration rate; EFVi, indexed epicardial fat volume; TR, tricuspid regurgitation

^*^P < 0.05

No statistically significant correlations were observed between EFVi and other target organ damage parameters after controlling for age. Specifically, EFVi was not significantly associated with eGFR (r = 0.120, P = 0.308), urine ACR (r = − 0.131, P = 0.413), baPWV (r = 0.145, P = 0.218), ABI (r = 0.167, P = 0.153), or TR velocity (r = − 0.026, P = 0.877).

In the multivariable model predicting septal e′ velocity, EFVi demonstrated a significant independent association (β = –0.0003; 95% CI, –0.0005 to –0.0001; P = 0.007). Similarly, for the E/e′ ratio, EFVi showed an independent association (β = 0.0606; 95% CI, 0.0093 to 0.1120; P = 0.024). These findings indicate that the relationships between EFV and LV diastolic function parameters persisted even after accounting for traditional cardiovascular risk factors and renal function.

### ROC analysis for predicting diastolic dysfunction

**Fig. 3:**
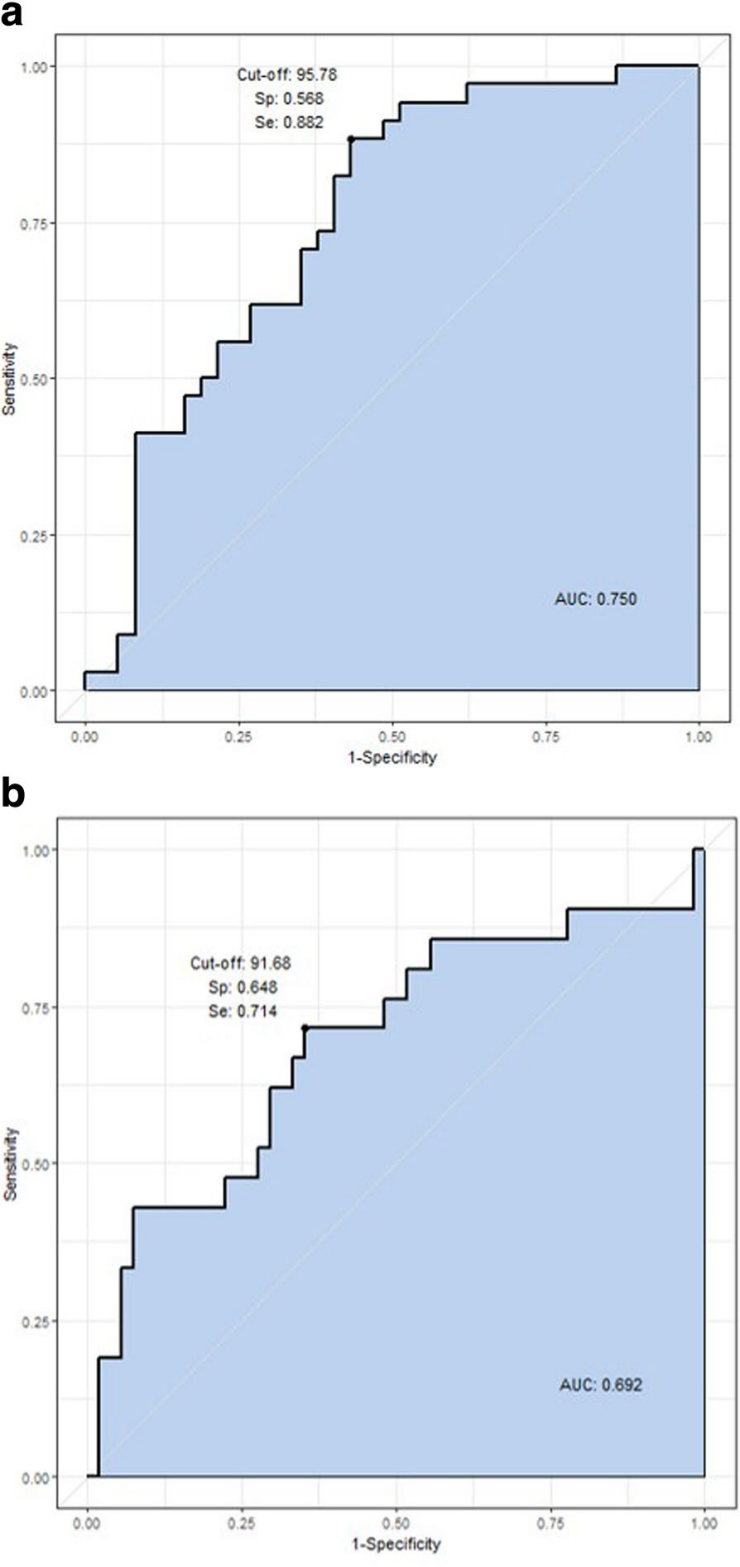
Receiver operating characteristic (ROC) curves of indexed epicardial fat volume (EFVi) for predicting left ventricular (LV) diastolic dysfunction. **A** The ROC curve for predicting septal e′ velocity < 7 cm/sec: area under the curve (AUC), 0.750; 95% confidence interval (CI), 0.640–0.860; sensitivity, 88.2%; specificity, 56.8%; cutoff, 95.78 cm^3^/m^2^. **B** The ROC curve for predicting an E/e′ ratio > 12: AUC, 0.692; 95% CI, 0.535–0.798; sensitivity, 71.4%; specificity, 64.8%; cutoff = 91.68 cm^3^/m^2^

To evaluate the diagnostic utility of EFVi in identifying patients with impaired LV diastolic function, ROC curve analyses were performed using clinically relevant cutoff values. Figure 3 presents the ROC curves for two different diastolic dysfunction thresholds.

For predicting septal e′ velocity < 7 cm/sec, which represents reduced early diastolic tissue velocity, EFVi showed superior diagnostic performance with an AUC of 0.750 (95% CI, 0.640 to 0.860). At the optimal cutoff value of 95.78 cm^3^/m^2^, the sensitivity was 88.2% and specificity was 56.8%.

For predicting an E/e′ ratio > 12, which indicates elevated LV filling pressures, EFVi demonstrated an AUC of 0.692 (95% CI, 0.535 to 0.798). The optimal cutoff value was 91.68 cm^3^/m^2^, yielding a sensitivity of 71.4% and specificity of 64.8%.

## Discussion

The present study investigated the association between EFVi measured by ECG-gated CT and target organ damage parameters in subjects with nonobstructive CAD. Our principal findings demonstrate that EFVi was significantly and independently associated with echocardiographic parameters of LV diastolic function, specifically septal e′ velocity and E/e′ ratio, even after adjusting for potential confounders including age, BMI, hypertension, diabetes mellitus, smoking status, and eGFR. Notably, EFVi was not significantly associated with other target organ damage parameters, including eGFR, ACR, baPWV, or ABI. These findings suggest that EAT may have a preferential impact on cardiac diastolic function, an association not seen in other manifestations of target organ damage.

The observed association between EFVi and LV diastolic dysfunction can be explained through several plausible pathophysiological mechanisms. Epicardial fat exerts local paracrine effects on the adjacent myocardium through the secretion of various bioactive adipokines, cytokines, and inflammatory mediators [1, 2]. Unlike other visceral fat depots, EAT is not separated from the myocardium by fascia, allowing direct molecular communication between adipose tissue and cardiac muscle [5]. This anatomical proximity enables EAT to influence myocardial metabolism, inflammation, and fibrosis through the release of pro-inflammatory cytokines such as tumor necrosis factor α, interleukin 6, and monocyte chemoattractant protein 1 [6].

Furthermore, increased EAT volume may contribute to LV diastolic dysfunction through mechanical effects, creating a physical impediment to ventricular filling and promoting increased ventricular stiffness [7]. The age-related redistribution of adipose tissue toward visceral compartments, including the epicardial space, may also play a role in the development of diastolic dysfunction through altered adipokine secretion profiles and increased oxidative stress [8]. Additionally, EAT-derived free fatty acids may directly infiltrate the myocardium, leading to lipotoxicity and impaired diastolic relaxation [9].

Our findings are consistent with previous studies demonstrating associations between EAT and LV diastolic dysfunction. Fenk et al. [11] reported that successful weight reduction in severely obese patients improved LV diastolic function, suggesting a mechanistic link between adipose tissue and cardiac performance. Similarly, Turak et al. [12] found that increased echocardiographic epicardial fat thickness and high-sensitivity C-reactive protein levels were associated with diastolic dysfunction in patients with newly diagnosed essential hypertension.

Studies by Kim et al. [15, 18] have consistently demonstrated relationships between body fat parameters and cardiovascular outcomes. Kim et al. [18] showed significant associations between arterial stiffness and LV diastolic function in relation to gender and age, supporting the concept that vascular and cardiac target organ damage may share common pathophysiological pathways. Additionally, their work on body fat parameters and arterial stiffness provides important context for understanding how adipose tissue distribution affects cardiovascular health [15].

The use of advanced imaging techniques for EAT quantification has been validated in recent investigations. West et al. [4] demonstrated the clinical utility of deep learning approaches for EAT assessment with CT, highlighting implications for cardiovascular risk prediction. This technological advancement supports the reliability of our EFV measurements on CT using deep learning-based semiautomatic segmentation.

While our study found significant associations between EFVi and LV diastolic function, some investigations have reported conflicting results regarding EAT's relationship with cardiovascular parameters. Nelson et al. [13] questioned whether epicardial fat represents an additional measurement for subclinical atherosclerosis and cardiovascular risk stratification, suggesting that its clinical utility may be limited in certain populations. These discrepancies may be attributed to differences in imaging modalities, with echocardiographic thickness measurements potentially being less accurate than volumetric CT assessments.

Population characteristics may also influence the relationship between EAT and cardiovascular outcomes. Studies in different ethnic groups or those with varying degrees of cardiovascular risk may yield different results due to genetic factors, lifestyle differences, or varying distributions of traditional risk factors [10]. Additionally, methodological variations in EFV quantification, including different software platforms and segmentation approaches, may contribute to inconsistent findings across studies.

Our study addresses several important research gaps in the field of cardiovascular imaging. The use of deep learning-based semi-automated segmentation through 3D U-Net technology represents a significant methodological advancement over traditional manual or semiautomated approaches. This artificial intelligence-driven quantification method provides enhanced accuracy and reproducibility for EFV measurement, potentially improving the clinical utility of this biomarker [20].

Few prior studies have comprehensively assessed EFVi in relation to multiple target organ damage parameters within a well-characterized population of subjects with nonobstructive CAD using ECG-gated CT. The focus on indexed EFV, normalized for BSA, provides a more standardized metric for comparison across individuals with different body sizes. Additionally, the systematic evaluation of diverse target organ damage manifestations, including renal function, arterial stiffness, and cardiac diastolic function, offers insights into the preferential associations of EAT with specific organ systems.

The strengths of our investigation include the prospective study design, which minimizes selection bias and enables systematic data collection. The use of advanced imaging techniques, including ECG-gated CT with deep learning–based semiautomated segmentation, provides accurate and reproducible EFV measurements. The comprehensive assessment of multiple target organ damage parameters allows for comparative evaluation of EAT's associations with different organ systems. Additionally, the strict inclusion criteria ensuring subjects had nonobstructive CAD helps isolate the effects of EAT from confounding influences of significant coronary stenoses.

Several limitations should be acknowledged. The single-center design may limit the generalizability of our findings to other populations or healthcare settings. The relatively small sample size of 75 subjects may have limited statistical power to detect smaller associations, particularly with other target organ damage parameters. Potential residual confounding from unmeasured variables cannot be entirely excluded despite multivariable adjustment. The cross-sectional nature of the study precludes determination of causality or assessment of long-term prognostic implications. Finally, the lack of long-term follow-up data limits our ability to evaluate the clinical significance of the observed associations.

## Conclusions

This study demonstrates that EFVi measured by ECG-gated CT with deep learning–based semiautomated segmentation was independently associated with echocardiographic indicators of LV diastolic dysfunction in subjects with nonobstructive CAD. The preferential association with cardiac diastolic function, which was not found in other manifestations of target organ damage, suggests that EFVi may serve as a valuable imaging biomarker for early detection of cardiac target organ damage. These findings support the potential clinical utility of EFVi as a tool for cardiovascular risk stratification and early intervention strategies.

## Supplementary Information

Supplementary Material 1. Fig. S1. Correlation between age and indexed epicardial fat volume (EFVi).

## Data Availability

The datasets generated and/or analyzed during the current study are not publicly available due to patient privacy restrictions but are available from the corresponding author on reasonable request.

## Abbreviations

ABI: Ankle-brachial index
ACR: Albumin to creatinine ratio
AUC: Area under the receiver operating characteristic curve
baPWV: Brachial-ankle pulse wave velocity
BMI: Body mass index
BSA: Body surface area
CAD: Coronary artery disease
CCTA: Coronary computed tomography angiography
CI: Confidence interval
CT: Computed tomography
EAT: Epicardial adipose tissue
ECG: Electrocardiogram
EFV: Epicardial fat volume
EFVi: Indexed epicardial fat volume
eGFR: Estimated glomerular filtration rate
HbA1c: Hemoglobin A1c
HU: Hounsfield units
LV: Left ventricular
LVEF: Left ventricular ejection fraction
ROC: Receiver operating characteristic
TR: Tricuspid regurgitation
TTE: Transthoracic echocardiography
