# Supplementary material for: Association of deep learning–derived epicardial fat volume with target organ damage in subjects with nonobstructive coronary artery disease
Source: J Cardiovasc Imaging. 2025 Dec 25;33:17. doi: 10.1186/s44348-025-00062-5 (PMC12739840; doi:10.1186/s44348-025-00062-5)

Supplementary Figure 1. Correlation Between Age and Indexed Epicardial Fat Volume (EFVi)

Scatter plot showing a moderate positive linear correlation between age and EFVi (r = 0.567, P < 0.001). The red line represents a smoothed trend line derived from local regression. EFVi, indexed epicardial fat volume.


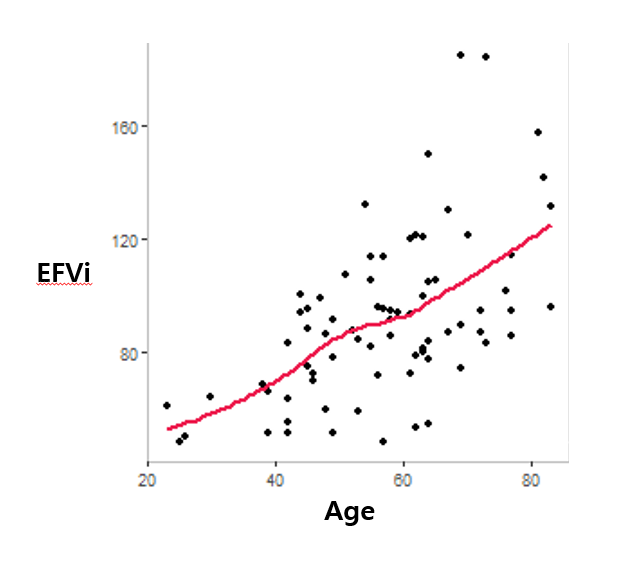

Supplement: Supplementary file 1 — Supplementary Material 1. Fig. S1. Correlation between age and indexed epicardial fat volume (EFVi). [file 44348_2025_62_MOESM1_ESM.docx]
